# Commentary: Chloride Regulation: A Dynamic Equilibrium Crucial for Synaptic Inhibition

**DOI:** 10.3389/fncel.2016.00182

**Published:** 2016-07-19

**Authors:** Staffan Johansson, Tushar D. Yelhekar, Michael Druzin

**Affiliations:** Section for Physiology, Department of Integrative Medical Biology, Umeå UniversityUmeå, Sweden

**Keywords:** chloride, Cl^−^ channel, K^+^ Cl^−^ cotransporter 2, conductance, membrane potential, synaptic inhibition, Cl^−^ concentration, equilibrium potential

The recent review by Doyon et al. (2016) is for the main part an excellent description of many important aspects of neuronal chloride regulation and will be of good use to many scientists interested in synaptic function. Nevertheless, some information given on the role of the K^+^ Cl^−^ cotransporter 2 (KCC2) is likely to be misleading. While proposing an explanation for the controversial findings by Glykys et al. (2014) that blockers of cation-chloride cotransporters did not affect the basal intracellular Cl^−^ concentration ([Cl^−^]_i_), contrary to expectations from previous transporter manipulations (see e.g., references in Ben-Ari, 2014; Kaila et al., 2014), Doyon et al. (2016) suggest that this may be due to the small degree of inhibitory synaptic activity in the preparation used, with extremely low Cl^−^ load. In their explanation, they give an equation for the equilibrium relation between Cl^−^ flux through an inhibitory (Cl^−^) conductance (g_inh_) and the Cl^−^ transported by KCC2. On basis of this equation, the conclusion is that the Cl^−^ equilibrium potential “E_Cl_ is sensitive to changes in KCC2 activity (g_KCC2_) only when Cl^−^ load (g_inh_) is large.” This conclusion, however, cannot be justified on basis of the relation between Cl^−^ flux through channels and Cl^−^ transported by KCC2. (The equation given by Doyon et al. is not correctly formulated, although the reason for their claim may not depend on this mistake).

For an explanation of our point of view, consider a hypothetical cell with Cl^−^ transport across the outer membrane only via Cl^−^ selective channels and KCC2. At equilibrium, the amount (mol/s) of Cl^−^ transported by the channels must be equal, but opposite, to that transported by KCC2. We thus formulate the relation:

(1) $${\text{I}}_{\text{Cl}}/\text{F }={\text{ g}}_{\text{KCC}2}{\text{U}}_{\text{KCC}2}$$

where I_Cl_ is Cl^−^ current, F is the Faraday constant and U_KCC2_ is the driving force for transport by KCC2. g_KCC2_ is a proportionality factor that may be thought of as an “apparent conductance,” and should reflect the number of transporters in the membrane as well as the transport rate of the individual transporter molecules at fixed K^+^ and Cl^−^ concentrations, similarly as I_Cl_ depends on the Cl^−^ conductance (g_Cl_) which reflects the number of Cl^−^ channels as well as the conductance of individual channels. (g_KCC2_ is, however, not a conductance in the usual electrical sense). Equation (1) may be reformulated, in several steps, for clarity:

(2) $${\text{g}}_{\text{Cl}}\;({\text{V}}_{\text{m}}-{\text{E}}_{\text{Cl}})/\text{F}={\text{g}}_{\text{KCC}2}\;(\text{RT ln }({\left[{\text{Cl}}^{-}\right]}_{\text{i}}/{\left[{\text{Cl}}^{-}\right]}_{\text{o}})+\text{RT ln }({\left[{\text{K}}^{+}\right]}_{\text{i}}/{\left[{\text{K}}^{+}\right]}_{\text{o}}))$$

(3) $${\text{g}}_{\text{Cl}}\;({\text{V}}_{\text{m}}-{\text{E}}_{\text{Cl}})/{\text{F = g}}_{\text{KCC}2}\text{ F }({\text{E}}_{\text{Cl}}-{\text{E}}_{\text{K}})$$

(4) $$\begin{array}{l} {\text{E}}_{\text{Cl}}=({\text{g}}_{\text{KCC}2}\;{\text{F}}^{2}\;{\text{E}}_{\text{K}}+{\text{g}}_{\text{Cl}}{\text{V}}_{\text{m}})/\\ \;({\text{g}}_{\text{KCC}2}\;{\text{F}}^{2}+{\text{g}}_{\text{Cl}}) \end{array}$$

where V_m_ is membrane potential, R the gas constant, T temperature (in K), E_K_ the K^+^ equilibrium potential and Cl^−^ and K^+^ concentrations are given within brackets with subscripts i and o for inside and outside, respectively.

We may use Equation (4) to illustrate the relation between E_Cl_ and g_KCC2_ at various levels of g_Cl_. (Assume that K^+^ concentrations and membrane potential are fixed, as controlled by other factors, such as the cellular Na^+^-K^+^-ATPase, not discussed here). As can be seen in Figure 1A, contrary to the claim by Doyon et al. (2016), E_Cl_ is only weakly dependent on KCC2 transport capacity at high Cl^−^ conductance, while it depends strongly on KCC2 when Cl^−^ conductance is low. At the extremes, when g_KCC2_ = 0, then E_Cl_ = V_m_ and when g_Cl_ = 0, then E_Cl_ = E_K_.

**Figure 1:**
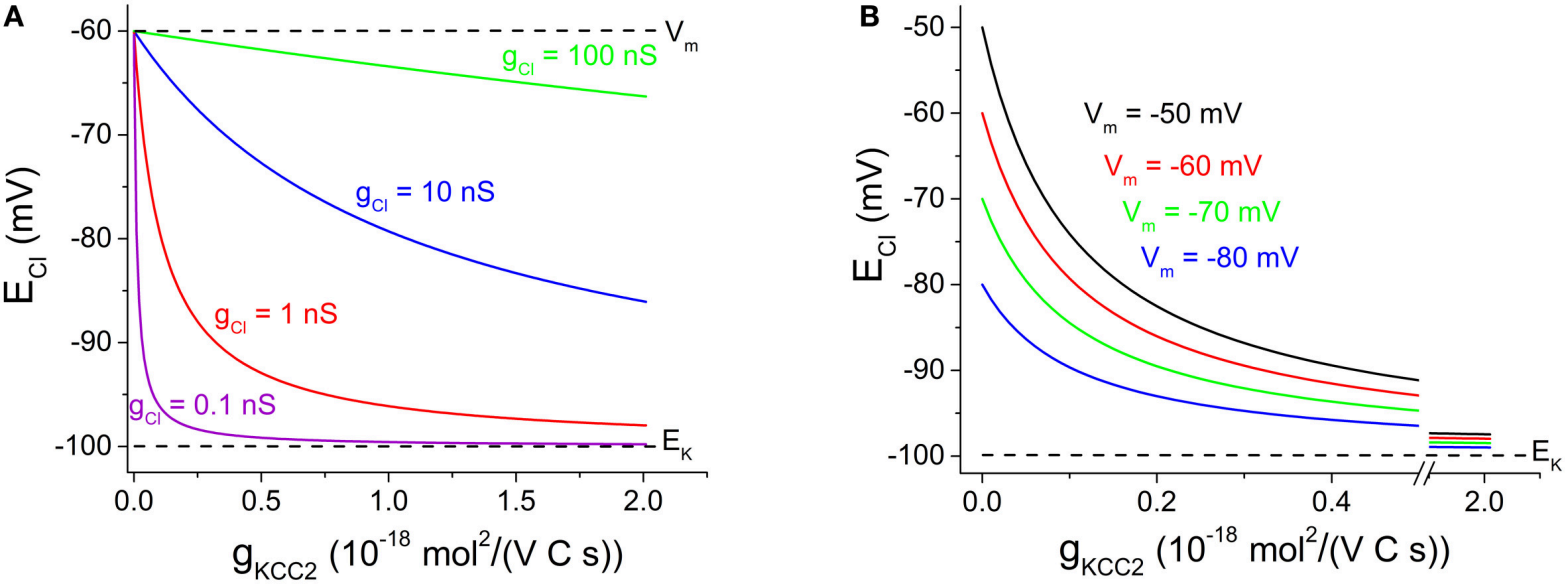
**Cl^−^ equilibrium potential — dependence on KCC2 transporter capacity at various Cl^−^ conductance and various membrane potentials. (A)** E_Cl_ vs. g_KCC2_ with E_K_ fixed at −100 mV and V_m_ at −60 mV. g_Cl_ as indicated. Note that E_Cl_ dependence on g_KCC2_ is reduced with increased g_Cl_. **(B)** E_Cl_ vs. g_KCC2_ with E_K_ fixed at −100 mV and g_Cl_ at 1 nS. V_m_ as indicated. Note that E_Cl_ dependence on g_KCC2_ increases when V_m_ changes in positive direction. Note the x-axis break between 0.5 and 1.9 10^−18^ mol^2^/(V C s), to clearer illustrate the steeply decaying region of the curves. Justification of illustrated parameter ranges: The g_Cl_ range (in **A**) was chosen to include cells with a low g_Cl_ as evident from the high membrane resistance (Johansson et al., 1995) as well as cells with a high g_Cl_ (very low input resistance dominated by inhibitory conductances; Destexhe et al., 2003). The g_KCC2_ range (in **A,B**) shown likely covers the capacity for most central neurons: When g_KCC2_ = 1 10^−18^ mol^2^/(V C s), KCC2-mediated transport modeled as described by Karlsson et al. (2011) may reduce [Cl^−^]_i_ from 20 mM to ~5 mM with approximated time constants of 0.85, 6.8, and 55 s for spherical cells of radius 5, 10, and 20 μm, respectively, assuming 50% cytosolic volume and no other Cl^−^ transport/leak. Experimentally observed [Cl^−^]_i_ recovery is slower or comparable (Berglund et al., 2006; Lee et al., 2011; Pellegrino et al., 2011).

The relation between E_Cl_ (and thus [Cl^−^]_i_) and transporter capacity has some bearing for the interpretation of the controversial findings by Glykys et al. (2014). Contrary to the suggestion by Doyon et al. (2016), Figure 1A shows that transporter block is expected to affect basal [Cl^−^]_i_ especially under conditions when g_Cl_ is low. Thus, other explanations than a low g_Cl_ must be sought for the controversial findings of Glykys et al. (2014). An *increased* g_Cl_, perhaps due to increased non-specific leak, could in theory contribute to the limited effect of KCC2 blocker on [Cl^−^]_i_.

On the other hand, the lack of *excitatory* synaptic input may contribute to a reduced sensitivity to transport block. In equation (4) above, a steady excitatory input may be represented simply by a more positive V_m_, if we assume that E_K_ is still maintained (by the cellular Na^+^-K^+^-ATPase). Figure 1B shows that although E_Cl_ is more positive, the dependence of E_Cl_ on g_KCC2_ is clearly stronger at more positive V_m_.

As described, an increased g_Cl_ (Figure 1A) or a more positive V_m_ (Figure 1B) will change E_Cl_ in positive direction. This may be exploited experimentally e.g., by combining GABA or glycine application with depolarization to achieve a dramatic change in E_Cl_ and rise in [Cl^−^]_i_ (Karlsson et al., 2011). With such manipulations, it is obvious that the neuronal transporter capacity cannot prevent the changes in E_Cl_ and in steady-state [Cl^−^]_i_.

Doyon et al. (2016) also note the neglected problem of apparent (illusory) conductance decrease based on recordings at different holding potentials when [Cl^−^]_i_ is changing, a problem which has recently been described in more detail by Yelhekar et al. (2016). It may be noted that experimentally, the effects of a changing [Cl^−^]_i_ on apparent conductance may be separated from true changes in conductance by using rapid voltage-ramp techniques (Karlsson et al., 2011; Yelhekar et al., 2016).

## Author contributions

SJ made the computations and paper writing. SJ, TY, and MD contributed to the ideas and final content.

### Conflict of interest statement

The authors declare that the research was conducted in the absence of any commercial or financial relationships that could be construed as a potential conflict of interest.
